# Immunosenescence and inflammaging in the aged horse

**DOI:** 10.1186/s12979-022-00325-5

**Published:** 2023-01-06

**Authors:** Sally DeNotta, Dianne McFarlane

**Affiliations:** Department of Large Animal Clinical Sciences, College of Veterinary Medicine, University of Florida, Gainesville, FL USA

**Keywords:** Adaptive immunity, Aging, Equine, Geriatric horse, Immune dysfunction, Innate immunity, Proinflammatory

## Abstract

The equine population in the United States and worldwide now includes a higher percentage of geriatric horses than ever previously recorded, and as methods to treat and manage elderly equids are developed and refined, this aging population will likely continue to expand. A better understanding of how horses age and the effect of age on immunity and disease susceptibility is needed to enable targeted preventative healthcare strategies for aged horses. This review article outlines the current state of knowledge regarding the effect of aging on immunity, vaccine responsiveness, and disease risk in the horse, highlighting similarities and differences to what is observed in aged humans. Horses show similar but milder age-related alterations in immune function to those reported in people. Decreases in lymphocyte proliferation and antibody production and diminished response to vaccination have all been documented in elderly horses, however, increased risk of infectious disease is not commonly reported. Aged horses also show evidence of a proinflammatory state (inflammaging) yet appear less susceptible to the chronic diseases of people for which inflammation is a risk factor. Information is currently lacking as to why the horse does not experience the same risk of age-related disease (e.g., cancer, heart disease, neurodegeneration) as people, although a lack of negative lifestyle habits, differences in diet, exercise, genetics and physiology may all contribute to improved health outcomes in the older horse.

Similar to demographic trends reported in both human and pet populations, the percentage of horses considered “aged” has been increasing, with estimates ranging from 22- 34% of all horses being older than 15 years of age [1, 2]. In the United States, the number of senior horses (over 20 years) has more than doubled since 1998, and has increased by 50% in the past decade [3]. This trend is likely due to improvements in preventative healthcare as well as an increasing societal view of horses as companion animals as opposed to livestock. Routine vaccination, endoparasite control and preventative dentistry have undoubtedly been the largest contributors to improved horse health and longevity in the last century. Of particular interest in geriatric equine healthcare are methods to support and bolster effective immunity to infections while reducing incidence of inflammatory disease.

Immunosenescence is the process of immune dysfunction that occurs in humans and animals as they age, and is an important risk factor for morbidity and mortality in people. It contributes to the development of most of the chronic health conditions that affect the elderly, including cancer, heart disease, inflammatory and degenerative diseases. Furthermore, decline in immune competency is a primary contributor to the increased susceptibility of the aged to infectious pathogens and a decreased effectiveness of vaccines to protect against infections commonly observed in aged people [4, 5].

Similar to humans, age-related alterations in immune function occur in horses (Table 1). In people, age-associated changes to the immune system affect both innate and adaptive immunity, and include alterations in the composition of lymphocyte populations, immune response to provocation, and a generalized proinflammatory state [6]. Diminished ability to respond to pathogen challenge is also observed in aged horses, but appears milder than that observed in people and specific modifications in vaccination schedule for older horses are not currently recommended. In addition, an effect of old age on risk of infectious disease has not been well documented, and very few of the most important infectious diseases in equids have increasing age as a risk factor for disease incidence or severity. It is unclear what factors are responsible for the milder age-related decline in immune function observed in horses, but the absence of known lifestyle risks for human chronic disease (such as excessive alcohol consumption, smoking, lack of physical exercise or poor diets) as well as differences in physiology are likely beneficial in the aging equid. While response to provocation appears only mildly impaired in aged horses, an enhanced inflammatory reactivity known as inflammaging, may be more of a concern in aged horses. Inflammaging may contribute to diseases common in aged equids, such as degenerative joint disease and reactive airway disease [7, 8]. Despite experiencing an age-related proinflammatory state, senior horses are far less prone to the leading causes of mortality in the aged human population, such as cancer, heart disease, cerebrovascular disease, chronic lower respiratory diseases and Alzheimer’s disease, all of which have been linked to inflammaging [9]. Understanding these differences and similarities in aging between the species could prove informative for horses and humans alike. This article reviews the current state of knowledge regarding interactions of age on immunity, vaccine responsiveness, and disease risk in the horse, highlighting similarities and differences to immunosenescence processes observed in aged humans.

**Table 1:** Summary of age- associated immune changes in horses versus humans

		**Horse**	**Human**
**A. Lymphocyte Alterations**			
Total lymphocyte Count		↓	↓
B-cell count		↓	↓
CD4 and CD8 count		↓	↓
CD4:CD8		↑	↑
FOXP3 + CD4 + (T-regulatory) count		↓	↓
Proliferation		↓	↓
B. **Neutrophil Alterations**			
Neutrophil count		NC	NC
Adhesion		NC	↑
Phagocytosis		NC	↓
Chemotaxis		↑	↓
Oxidative Burst		NC	↓
C. **Unstimulated Cytokines**			
TNF-α expression		↑	↑
IL-6 expression		↑	↑
IL-8 expression		↑	↑
IFN-γ expression		↑	↑
IL-1β expression		↑	↑
Serum TNF-α		↑	↑
**D. Cytokine Responsiveness**			
	**Stimulant**		
TNF-α	LPS, PMA, ionomycin	↑	↑
IFN-γ	LPS, PMA, ionomycin	↑	↑

## Special considerations for immunosenescence research in horses

Studies of immunosenescence in horses are complicated by a unique set of confounders, and as a result, study conclusions are often contradictory. For example, the definition of “aged” is often arbitrary in equine research, making comparisons across studies difficult. If subjects are too young, aging changes may not yet have occurred. If too old, there may be a bias towards including those animals with exceptional immune function [10]. In aged horses of unknown age, dental wear is often utilized as a method for age approximation. This method is particularly useful for young horses still undergoing the transition from deciduous to permanent teeth, but becomes highly inaccurate in aged horses, in whom diet, veterinary dental care, and genetics may all have substantial roles in overall dental wear [11]. The recent development of ‘epigenetic aging clocks’ in humans, mice, and equids offers a novel method for determining relative biologic age. The process of epigenetic aging uses methylation arrays to profile large numbers of CpG positions in the genome, and in human tissue samples is predictive of mortality even after adjusting for known risk factors such as chronological age, sex, smoking status, and other comorbidities [12, 13]. Epigenetic aging clocks have been developed for horses, and may offer a quantifiable method to determine relative age in individuals, compare the age-accelerating effects of common geriatric diseases, and evaluate the efficacy of interventions designed to prevent or delay immunosenescence in aged horses [14, 15].

In addition to difficulties related to defining and determining age in geriatric horses, identifying specific causes of age-induced decline in immune function is challenging. Ruling out the presence of subclinical disease is necessary but difficult in an aged population [16]. In older horses, the high prevalence of co-morbidities that contribute to chronic, low-grade inflammation may affect studies on immunosenescence. For example, osteoarthritis or reactive airway disease, two of the most common diseases in age horses, have been largely ignored in studies of aging in horses thus far. Furthermore, endocrine and metabolic abnormalities common to old horses can strongly influence immune function. Equine pituitary pars intermedia dysfunction (PPID), a disease that affects 15–30% of horses > 20 years of age, is immunosuppressive, while equine metabolic syndrome (EMS) an equally common condition, is proinflammatory [17–20]. Ideally a strict inclusion protocol should be employed when enrolling participants into studies of aging immunity. In human immunological research, procedures have been developed [21], which combine history, clinical examination, and diagnostic laboratory results to recruit healthy aged subjects for study inclusion. Adoption of similar screening protocols may improve the quality of data gathered and the concordance among multiple studies in veterinary gerontology. Additionally, in equine studies, one must consider hormonal status throughout the study period, as pituitary and adrenal hormones are strong modifiers of immune function and are also often affected by common equine geriatric disorders. The marked effect of season on the output of anti-inflammatory hormones from the pituitary gland and the high prevalence of pituitary dysfunction (PPID) in the aged equine population [22–25] can greatly confound studies of age-related immune function in horses. Obesity is known to affect immunity and inflammation in horses, and failure to control for body condition score can obscure the interpretation of results [26–28]. Other potential confounders when assessing immune function in aged horses include diet, supplements, medications, exercise, travel, and environmental exposures of the study participants.

## Age-associated changes in equine immune function

### Cell populations

Alterations in lymphocyte subset populations as a function of age have been documented in several species including people, dogs, and rodents [29–32]. Changes documented across species include a decrease in the number of naïve T cells (CD45RA), which has been documented in multiple species [33–37]. The decrease in naïve T cells may be the result of thymic involution or alternatively. the consequence of chronic antigenic stimulation [38–41]. In particular, chronic infection with cytomegalovirus (CMV) is thought to play a role in the depletion of the naïve lymphocyte pool [42]. Concurrent with loss of naïve T cells, clonal expansion of cytomegalovirus-specific CD8 memory cells has been observed in elderly primates [41–43]. The theory that exposure to pathogens can restructure the immune system even in the absence of clinical disease is supported by studies of CMV infection in specific-pathogen-free mice, where CMV infection resulted in reduced T cell responses and vaccination efficiency as well as accelerate accumulation of effector memory CD8 T cells [44, 45]. At this time, the lack of antibodies capable of differentiating equine naïve T-cells from memory T-cells has hindered studies investigating age-associated changes to T cell populations in the horse.

Other findings in human leukocyte populations include alterations in the total number of lymphocytes, CD4, CD8, T- regulatory cells, and B-cells as well as CD4:CD8 ratio [46–48]. Several studies have confirmed similar findings in aged horses and ponies [49–54]. The total number of lymphocytes, CD4, CD8, T-regulatory cells, and B cells all decrease in aged horses [49–54] while CD4:CD8 ratio, a proinflammatory marker, increases in aged equids [51, 52]. The percentage of FOXP3 + CD4 + cells (T-regulatory cells), is also decreased in horses over 15 years of age [54]. T-regulatory cells have an anti-inflammatory role, thus the loss of this cell type with age further drives a proinflammatory phenotype.

While little work has been done in horses examining age-related changes to lymphoid organs such as the thymus, spleen, and lymph nodes, the widespread use of regenerative therapies in equine practice has led to multiple studies comparing stem cell populations in adult and geriatric horses. Studies of equine stem cells derived from bone marrow, adipose, and synovial fluid have demonstrated progressive reductions in cell proliferation and differentiation potential, as well as increased prevalence of age-related binucleate or tetraploid cells. Additionally, stem cells derived from geriatric horses are more likely to show morphological features correlated with aging such as endoplasmic reticulum stress, autophagy, and mitophagy [55–58].

### Innate immunity

Although immunosenescence predominantly impairs T cell function, changes in innate immunity occur as well. Innate immunity is considered the first line of defense and includes soluble mediators as well as phagocytic effectors cells such as neutrophils and macrophages. Across species, alternations in neutrophil function associated with increasing age include reductions in phagocytosis, oxidative burst, chemokinesis, and chemotaxis [59–62]. In contrast, adhesion was found to be greater in neutrophils collected from people 65–80 years old, with increased expression of adhesion molecule CD11b [63]. While most studies have not found a change in neutrophil numbers in the aged [59–62], Liu and colleagues [64] reported reduced neutrophil counts and functional alternations in immunity in extremely old female subjects, while Fernandez-Garrido and others [65] associated neutrophil count increases with frailty. Similarly, age-associated alterations in neutrophil function is associated with increased susceptibility to pseudomonas pneumonia infection in rodents [66]. In this same study, although concentrations of pulmonary chemokines were higher in old mice, neutrophil count in the airways was markedly lower following pseudomonas infection, suggesting a failure in neutrophil chemotaxis with age [66]. In contrast, a study of healthy aged horses found that neutrophil adhesion, oxidative burst, and phagocytosis were all similar to that of healthy adult horses, while chemotaxis was increased in aged horses compared to adult horses [67]. More recently, Miller et al. found that healthy aged horses had reduced concentrations of plasma myeloperoxidase (MPO) (a marker of neutrophil degranulation) when compared to healthy adult horses, with no differences in absolute numbers of segmented and band neutrophils or monocytes to otherwise account for the difference [68]. Differences in study design may explain the contrast in findings. The first study (McFarlane) assessed washed neutrophils stimulated ex vivo while the second (Miller) measured in situ (plasma) products of neutrophil response. The Miller group also reported increased TNFα expression after in vitro stimulation of whole blood with heat-inactivated *R. equi* in geriatric horses when compared to adult horses [68]. More recently, a study examining the effect of age on equine monocyte function and pro-inflammatory cytokine responses to bacterial lipopolysaccharide (LPS) found that similar to aged people, geriatric horses had reduced monocyte phagocytic capacity as well as increased IL-1β gene expression in response to lipopolysaccharide stimulation [26, 69]. The significance of these changes in innate immunity on disease susceptibility in the aged horse remains unclear.

In contrast to healthy aged horses, current evidence suggests that aged horses with PPID experience impaired neutrophil function and increased frequency of bacterial infection [67]. At this time, early detection of PPID is challenging, and it may therefore be prudent to assume all aged horses are at higher risk for neutrophil impairment until endocrine dysfunction can be definitively ruled out or until more sensitive early disease detection methods are available.

#### Cytokine and acute phase protein profiles in the aged horse

Serum cytokine profiles in aged people typically favor a pro-inflammatory phenotype [70, 71]. This process is known as inflammaging, and has been postulated to be a contributing mechanism in the process of immunosenescence and development of chronic age-related disease [72]. Aged horses also show similar cytokine profiles with increased gene expression of TNF-α, IL-6, IL-1β IL-8, IFN-γ, IL-15 and IL-18 [19, 52] and increased proinflammatory: anti-inflammatory cytokine ratios including IL6: IL10 and TNF-α:IL10 [19]. When cytokine concentration was examined at the protein rather than gene expression level, the findings were not as clear. Serum cytokine concentration of TNF-α was increased in aged horses in one study [52] but not another [19], although both studies were hindered by small sample size. In horses, serum TNF-α concentration can be affected by multiple confounding factors, including concurrent illness, obesity, inflammation and season, any of which could have played role in the contradictory findings in the studies. The role of other serum cytokines in geriatric horses has not been extensively examined.

Less data are available regarding the role of aging on acute phase protein concentration. Zak et al. reported serum amyloid A (SAA), c reactive protein (CRP), haptoglobin, activin A, α-1-antichymotrypsin, and procalcitonin did not differ between healthy adult and aged horses, however, as is common in equine studies, there was an insufficient sample size to draw conclusions without additional work to confirm the finding [17].

#### Immune responsiveness

Several studies have evaluated the impact of aging on equine peripheral blood mononuclear cells (PBMC) or whole blood response following ex vivo immune stimulation [19, 52, 53]. In aged primates, stimulation of whole blood or PBMC results in a greater release of proinflammatory cytokines than observed in adults [73–75]. Similarly, ex vivo stimulation of equine PBMCs revealed an increase in TNF-α and IFN-γ production with age [19, 52, 53]. This enhanced ex vivo cytokine response may not translate to cytokine response in natural disease, however. A study comparing inflammatory responses in adult and aged horses with naturally occurring intestinal disease found no differences in median concentration of type-2 cytokines IL-4 and IL-10 or type-1 cytokine IFN-γ [76]. Of note, inflammatory cytokines IL-6 and TNF-*α* were significantly higher in geriatric compared to young-adult horses at all sampling time points, corroborating previous studies suggesting a baseline proinflammatory phenotype in geriatric horses similar to that observed in people [19, 52, 53, 70, 71, 76].

### Adaptive immune function

#### Lymphocyte function

In studies of non-equid species, lymphocyte proliferation has been consistently found to decrease with age. While several mechanisms have been proposed to explain this age-related reduction in proliferation response, including decreases in serum IL-2 concentration and IL-2 receptor expression [35, 77, 78], lymphocyte impairment has also been observed in the absence of these two factors, suggesting that intracellular signaling defects may also be altered with age [79]. Similarly, horses also experience age-associated reductions in lymphocyte proliferation [50, 52]. The decrease in lymphocyte proliferation noted in geriatric horses does not appear to be responsive to IL-2 supplementation, nor does it seem to be associated with altered lymphocyte IL-2 receptor expression [50], suggesting that in the horse, altered intracellular signaling may be the primary mechanism behind age-related defects in lymphocyte division.

## Mechanisms of immunosenescence

Few studies have investigated the underlying mechanisms of immunosenescence in horses. Similar to humans, geriatric horses demonstrate age-associated alterations in leucocyte genomic stability; in one study, aged horses had increases in positive TUNEL cells, oxidative DNA damage, sister chromatid exchange and bleomycin-induced chromatid breaks when compared to non-aged adult horses [80]. Telomere length of leucocytes also decreases with age in horses, although the association between reduced telomere length and reductions in immune function in the elderly equine population have not been as clearly demonstrated as they have in aged people [81, 82]. Mitogen-induced proliferation of PBMCs was also shown to be weakly correlated to relative telomere length, leading the authors to suggest other mechanisms likely have a role in age-related decrease in PBMC proliferation [82]. This study also reported a positive correlation between telomere length and total IgG concentration as well as a negative correlation with inflammatory cytokine expression. Currently evidence that telomere length has a causative role in equine immunosenescence is lacking.

## Clinical consequences of immunosenescence in horses

### Infectious disease risk

The notion that geriatric horses are at increased risk for infectious diseases is commonly noted throughout the non-scientific equine literature, though there are few controlled studies to support this theory. Recently, trends in morbidity and mortality of aged horses have been investigated through a variety of epidemiological studies utilizing owner surveys, prospective studies with veterinary examinations, and analysis of medical records and pathology reports [83–87]. In all reports, infectious disease was an uncommon cause of disease and death in aged horses. Despite the suggestion that infectious conditions such as strangles (*Streptococcus equi equi*), influenza, or parasitism are more common in geriatric horses, to the authors’ knowledge, these assertions remain unsubstantiated.

While it appears that geriatric horses may not be broadly more susceptible to infectious disease, there are examples of specific pathogens with a predilection for aged horses. West Nile virus infection may cause more severe disease in geriatric horses compared to adults, as a higher case fatality rate has been reported in aged horses, particularly in previously unexposed animals [88, 89]. Similarly, in horses experimentally challenged with a strain of equine herpesvirus-1 (EHV-1) known to create neurologic disease, aged mares appear more susceptible to develop neurologic signs [90, 91]. In contrast, endoparasite egg shedding in horses does not appear to be affected by age. This observation was based on fecal egg count (FEC) before and after anthelmintic administration (measuring egg reemergence period and total FEC), not by the ideal method of performing worm counts at postmortem [92]. Unlike what is observed in healthy aged horses, two studies reported greater FEC and a shorter egg re-emergence period after anthelmintic treatment in horses with PPID [92, 93], while a group from Switzerland did not find higher FEC in aged horses with “pre-clinical PPID” [94]. Unlike the other 2 studies, they included only horses without clinical signs of PPID. Inclusion into the pre-clinical PPID group was based on ACTH concentration alone.

### Vaccine responsiveness

A key clinical feature of immunosenescence in humans is the progressive decline in antibody response to immunization. A failure to mount protective immunoglobulin concentrations to influenza is observed in the aged population, and contributes to the high morbidity and mortality due to influenza infection in older people [95, 96]. A diminished response of aged horses to influenza vaccination has also been corroborated in multiple studies [95, 97, 98]. Horohov, et al. reported aged ponies displayed a tenfold decrease in titers when compared to non-aged adults [99]. Using a different vaccine, Muirhead, et al. reported that although aged horses developed antibody concentrations considered to be protective, aged horses had lower concentrations of immunoglobulin subtypes IgGa and IgGb [95]. The immune response to influenza vaccination may be further altered in older obese horses and those affected by equine metabolic syndrome (EMS). A study comparing immune response to influenza vaccination in EMS and non-EMS horses resulted in similar humoral responses in both groups, but reduced cell-mediated immunity response in the EMS groups, with influenza-vaccinated EMS horses having lower gene expression of IFN-γ and IL-2 compared to vaccinated non-EMS control horses [100].

In practice, it is likely important to consider all possible confounders when developing a vaccination strategy for an aged horse, including general health, obesity, and potential endocrine disease. In one study of 200 aged horses, 26% were overweight (body condition score > 3/5) and 22% displayed hirsutism or delayed shedding suggestive of underlying PPID [101]. Despite substantial evidence that aged horses have altered immune response to influenza vaccination, there is a paucity of challenge studies investigating the risk of disease in geriatric horses following standard adult equine vaccination protocols for influenza or other infectious diseases. As a result, specific target titers for protecting aged horses from disease are currently unknown. It is important to note that despite observations of reduced effectiveness of influenza vaccine in aged horses, an increased incidence of naturally occurring influenza infection in older horses has not reported.

Immune response to naïve antigenic challenge has been specifically examined in horses greater than 20 years of age. Differing from what has been reported in other species [102–104], the magnitude of a primary antibody response in geriatric horses did not decline with age [95]. Following administration of rabies vaccine to naïve horses, antibody titers after both the first and second immunizations were not different between aged and non-aged adult horses. Of note, however, in this study 80% of the both the control and aged population were found to have low serum selenium concentrations, a potential study confounder. It is unclear if the low selenium may have resulted in a suboptimal vaccine response in both groups. Response to rabies immunization in aged horses was further studied by Harvey and colleagues [105]. They reported that horses > 20 years had a similar magnitude and duration of antibody response to adult horses, with an average duration of a protective titer of 2–3 years. In contrast, naïve horses did not reach protective titers after a single vaccination dose, irrespective of age. Further studies are needed to better characterize the effect of age on naïve and amnestic vaccine challenge and when needed, assist in the development of optimal vaccination protocol for geriatric horses. For the infrequent infectious agents where age appears to be a risk factor for more severe disease (West Nile Virus, EHV-1), of particular interest would be evaluating the use of adjuvants and high antigen dose, similar to those described for improving vaccination response in aged human patients [106], to improve vaccine efficacy in geriatric horses.

### Chronic inflammatory diseases

While aged horses generally maintain an adequate protective immunity against pathogens, the do exhibit a proinflammatory phenotype that may contribute to the high prevalence of inflammatory diseases observed in the geriatric equid. A recent study of 1448 horses greater than 20 years of age found 68.8% were affected with a chronic health condition, with osteoarthritis (42.4%), PPID (26.8%), dental disease (15.1%) and ophthalmic disease (11.1%) the most common conditions reported [107]. The specific mechanisms by which age-associated imbalances in cytokine and acute phase response contribute to the initiation or progression of chronic inflammatory diseases in older horses have not been elucidated, but in humans is postulated to be the result of increased concentrations of circulating proinflammatory mediators produced through chronic inflammatory stimulation, a process termed “inflammaging” [108, 109]. Studies of immune aging in humans have further revealed that oxidative stress has an important role in the development of chronic inflammatory disorders in aged people, likely due to an overall reduction in endogenous antioxidants to counterbalance the production of reactive oxygen species that are generated by many physiological cellular metabolic processes. Asthma, a syndrome of both humans and horses, is characterized by airway hyperresponsiveness, obstruction, mucus hyper-production, and airway wall remodeling, and increases in severity with age in both species. In horses, severe asthma is associated with a dysregulation of innate and acquired immunity resulting in neutrophilic inflammation and an overexpression of Th1, Th2, and/or Th17-type molecules [110]. In horses on pasture (a risk factor for the development of asthma) a significant age-related increase was found in the expression of IL-6, IL-8, TLR-4 and TNF-α in stimulated bronchoalveolar cells and for TNF-α in stimulated PBMCs, suggesting that both age and environment likely contribute to the development of disease [8].

Immunosenescence may also play an important role in geriatric horses’ ability to recover from inflammatory disorders. One study of horses with naturally-occurring colitis found the likelihood of non-survival increased by 11.8% for every year the horse aged, and that horses ≥ 20 years of age were 15.2 times more likely to die than young adults, independent of financial considerations, comorbidities, and duration of hospitalization [76]. In contrast, a study examining post-operative recovery from colic surgery in aged and adult horses found no differences in the severity of post-operative reflux or likelihood of survival [111]. Clearly much remains to be elucidated regarding the effect of age on disease in horses. A better understanding of the age-related immune dysfunction is needed to facilitate the development of effective preventative strategies to minimize chronic inflammatory diseases in old horses.

## Conclusions

The equine population in the United States and worldwide now includes a higher percentage of geriatric horses than ever previously recorded [3], and as methods to treat and manage elderly equids are developed and refined, this aging population will likely continue to expand. Similar to the aging population of people, much interest exists in maintaining optimal health and function well into late life, and strategies designed to preserve a youthful immune system in the old horse population are needed. In contrast to geriatric humans, healthy aged horses appear to be relatively effective at avoiding infectious diseases. Aged horses, however, do experience a high prevalence of immunosuppressive endocrine disease (eg., PPID), resulting in an increasingly large population of older horses at high risk of impaired immune function. Interventions designed to preserve adaptive immunity and deter the shift towards a proinflammatory bias that occurs in old horses may prevent or delay age-related comorbidities and promote equine health well into old age.

## Data Availability

Not applicable.

## Abbreviations

CMV: Cytomegalovirus
SAA: Serum amyloid A
CRP: C reactive protein
PBMC: Peripheral blood mononuclear cells
MPO: Myeloperoxidase
LPS: Lipopolysaccharide
PPID: Pituitary pars intermedia dysfunction
EHV-1: Equine herpesvirus-1
FEC: Fecal egg counts
EMS: Equine metabolic syndrome
